# Research on Time-Dimension Expansion of HBP Model Based on Hydroxyl-Terminated Polybutadiene (HTPB) Propellant Slurry

**DOI:** 10.3390/polym17121682

**Published:** 2025-06-17

**Authors:** Yanjun Bai, Jianru Wang, Yifei Feng, Peng Cao, Xiaorui Jiang

**Affiliations:** 1 Aerospace Commercial Rocket Power Technology Co., Ltd., Xi’an 710048, China; 2 Academy of Aerospace Solid Propulsion Technology, Xi’an 710025, China; 3 Aerospace Propulsion Technology Research Institute, Xi’an 710076, China; 4 College of Architecture and Civil Engineering, Beijing University of Technology, Beijing 100124, China; 5 School of Civil Engineering, Hebei University of Engineering Handan, Handan 056000, China

**Keywords:** viscoelastic fluid, propellant slurry, rheological test, rotary rheometer, viscosity constitutive mode

## Abstract

The curing reaction of hydroxyl-terminated polybutadiene (HTPB) solid propellant slurry alters its internal molecular structure, leading to variations in rheological properties. This study investigates the evolution of the rheological behaviour of HTPB propellant slurry during the curing process. Rheological parameters of the slurry at different curing stages were measured using a rotational rheometer, and its time-dependent rheological characteristics were systematically analysed. Building upon the Herschel–Bulkley–Papanastasiou (HBP) viscosity model, a temporal variable was innovatively incorporated to extend the model into the time domain, resulting in the development of the Herschel–Bulkley–Papanastasiou–Wang (HBPW) constitutive viscosity model. Model parameters were determined through experimental data, and the accuracy of the HBPW model was rigorously validated by comparing numerical simulations with experimental results. The findings demonstrate that the HBPW model effectively captures the viscosity variation patterns of HTPB propellant slurry with respect to both shear rate and curing time, exhibiting a minimal discrepancy of 1.7525% between simulations and experimental data. This work establishes a novel theoretical framework for analysing the rheological properties of HTPB propellant slurry, providing a scientific foundation for optimised propellant formulation design and processing techniques.

## 1. Introduction

The most important aspect of rheological research on solid composite propellant slurry is understanding the flow behaviour and deformation characteristics of the slurry selected. The interaction and dispersion of multiple components in a slurry determine its macroscopic rheological properties [1,2,3,4,5,6,7,8,9,10,11,12,13,14,15,16]. Studies have shown that varying the slurry formulation allows for effective control and adjustment of viscosity and yield stress [17,18,19,20,21,22,23,24,25,26]. Rheological tests typically include steady-state and dynamic shear tests, which allow important parameters like the viscosity–shear rate curve and slurry storage modulus to be determined. These parameters are critical in predicting the flow behaviour and stability of slurry under various shear conditions. As can be seen, rheological research can provide a theoretical foundation for the optimal design and process control of propellant slurry, improving the performance and reliability of solid rocket motors [27,28,29,30,31,32].

Hydroxyl-terminated polybutadiene (HTPB) propellant is widely used in the field of solid rockets, and the rheological properties of the propellant slurry are critical to ensuring the efficient and reliable operation of rocket engines [33,34,35]. It typically exhibits shear-thinning behaviour. At low shear rates, the propellant may behave viscoelastically, but at high shear rates, its viscosity decreases and fluidity increases.

Because the binder in the propellant component acts as a bridge between solid fillers, many researchers have studied its rheological properties. Researchers like Qian Zhang created a new type of energetic polymer adhesive called triblock copolymer polyNIMMO-HTPE-polyNIMMO by copolymerizing 3-nitromethyl-3-methyloxane (NIMMO) and hydroxyl-terminated polyether (HTPE). The polymerization approach increased the number of side groups and improved intermolecular interactions, resulting in a product with a higher viscosity that can be used as a propellant binder [6]. Researchers, such as Xiaomu Wen, introduced a modified hyperbranched polyester into an HTPE/AP/Al propellant and confirmed that it improved propellant performance [36].

The extremely high solid content of the composite solid propellant slurry leads to complex rheological properties. Several researchers have focused on improving the rheological properties of solid fillers. In 2019, Rohit Lade et al. discovered that incorporating aluminium nanoparticles into a composite solid propellant slurry significantly altered its rheological properties [37]. Following that, Rohit Lade et al. investigated the effects of aluminium nanoparticles on the rheological behaviour of an AP-based composite propellant slurry, conducted experimental research, and used mathematical modelling to predict and optimise propellant slurry rheology [38]. Lichen Zhang et al. investigated the dynamic layer-by-layer self-assembling method on the surface of aluminium fuel, which can improve propellant performance and combustion efficiency [39]. Rohit Lade et al. discovered that the addition of aluminium nanoparticles significantly improved the thermal–rheological properties of a hydroxyl-terminated styrene–butadiene rubber-based composite propellant slurry [40]. Yi Wang et al. discovered that increasing the sub-micron CL-20 content has a significant effect on the rheological properties of GAP propellant slurry [41]. Jing Zhang et al. investigated how varying hexamethylenediamine polymer (GAP) and aluminium powder contents affected the rheological properties of solid propellant slurry [42].

Pouring is an important process for applying propellant slurry that has caught the interest of some researchers. Haoyu Wang investigated and analysed the rheological properties and pouring process of hydroxyl-terminated styrene–butadiene rubber propellants. The results show that the flower board casting process has a significant impact on slurry cutting and separation [43]. With advancements in detection technology, methods such as infrared spectroscopy and X-ray scanning have been used to monitor the propellant pouring process. Weibin Wang and other researchers developed a method for tracking the mixing uniformity of HTPB propellant slurry using near-infrared (NIR) spectroscopy and orthogonal partial least squares discriminant analysis (OPLS-DA) [27]. Bo Jin et al. proposed a scheme that uses X-ray computed tomography (XCT) and the virtual element method (VEM) to create a mesoscopic viscoelastic model for solid propellants [44]. Other researchers have studied materials with similar rheological properties: Sridhar Viamajala investigated the slurry of corn straw and discovered that a higher solid level increases viscosity and yield stress [45]. Haiyu Sun’s team investigated gel propellants based on glycyrrhizic acid and discovered that hydrogels containing 1.0–2.0% glycyrrhizic acid exhibit shear-thinning behaviour at low shear rates [46].

This paper focuses on the rheological properties of HTPB propellant slurry during the curing process. The Herschel–Bulkley–Papanastasiou (HBP) viscosity model was extended in time to produce the Herschel–Bulkley–Papanastasiou–Wang (HBPW) viscosity model based on the rheological properties of the slurry. Rheological measurements of the propellant slurry were performed using a rotary rheometer, and the parameters of the HBPW viscosity constitutive model were obtained from test data. Simulation was used to validate the fitted model and confirm the accuracy of the HBPW viscosity constitutive model developed.

## 2. Viscosity Constitutive Model

The rheological measurement principle involves choosing a flow pattern and measuring the material functions of a fluid under different flow deformation conditions, including stress, strain, and strain rate variations. The constitutive model for viscosity involves establishing a correlation between fluid viscosity and shear rate. Below are several common constitutive models for viscosity. The model suitable for HTPB propellant slurry has been selected.

HTPB propellant slurry is a complex fluid with a variety of solid fillers and particles of different sizes. This results in multiple solid–liquid interfaces and dispersed structures within the solid-phase flow. The propellant slurry has a yield value, and the yield phenomenon in existing studies can only be described by Bingham fluid. Thus, the discussion below highlights the pros and cons of the Bingham model and its derived models.

In reality, many non-Newtonian fluids exhibit obvious yield phenomena. The power law viscosity model and other formulas are unable to accurately describe the yield phenomenon and the viscosity behaviour of fluid materials. They are empirical formulas and therefore lack precision. At the end of the twentieth century, scientists frequently referred to fluids with yield behaviour as Bingham fluids. This particular fluid requires external stress to surpass its yield stress in order to initiate flow.

The flow equation of the Bingham model can be expressed as follows:

(1) $$\gamma =\left\{\begin{array}{lll} 0 & & \tau <\tau_{y}\\ \left(\tau -\tau_{y}\right)/\eta & & \tau >\tau_{y} \end{array}\right.$$

where γ represents the shear rate of the fluid.

For certain Bingham fluids, they exhibit Newton’s viscosity law once they start flowing, as shown in Equation (2):

(2) $$\tau =\tau_{y}+\eta_{p}\gamma$$

where τ represents the shear stress on the fluid, $\tau_{y}$ represents the yield stress, and $\eta_{p}$ represents the viscosity after flow initiation.

**Figure 1:**
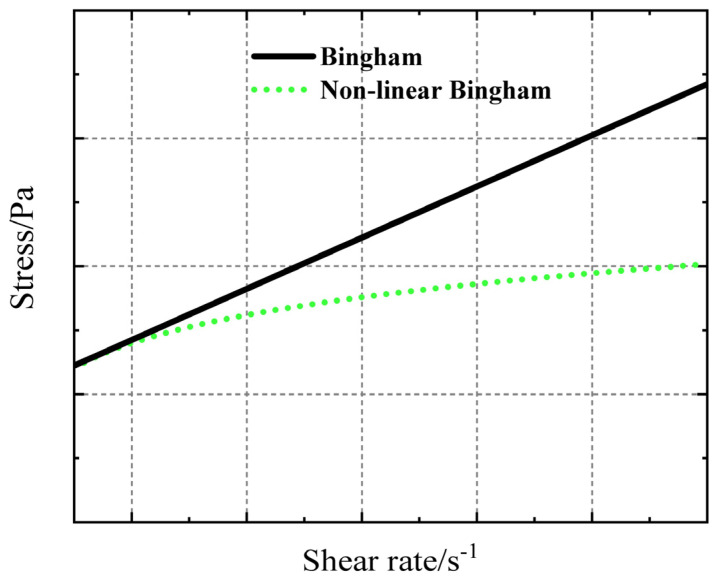
Shear stress curve of Bingham fluid.

The fluid is referred to as linear Bingham liquid or common Bingham liquid. Some Bingham liquids do not follow the Newton viscosity law, as the viscosity is affected by factors such as shear rate. This particular type of fluid is known as a non-linear Bingham liquid. Figure 1 displays the flow curves of the two fluids.

Consider that the flow pattern of the Bingham fluid follows a power-law model, that is,

(3) $$\tau =\tau_{y}+K\gamma^{n}$$

(4) $$\eta =\frac{\tau_{y}}{\gamma }+K\gamma^{n-1}$$

Under these circumstances, the material is known as Herschel–Bulkey fluid. The fluid properties described by the Herschel–Bulkely constitutive model include the following: the fluid has a yield value. When the fluid’s shear stress is less than the yield value, it does not flow; however, when the fluid’s shear stress exceeds the yield value, it flows, and its viscosity follows the power-law model. The properties of HTPB propellant slurry show that it has similar rheological behaviour, so this model is better suited to describing its rheological properties. When applied to engineering, researchers have proposed a number of modified models based on the Herschel–Bulkely constitutive model for various viscoelastic fluids.

Viscosity divergence is always encountered when calculating small and large deformations. To accurately describe the non-linear relationship between the shear stress and shear strain of viscoelastic fluid at large strain and avoid numerical divergence in calculating the effective viscosity coefficient using the Bingham model and its derivative Herschel–Bulkley model, the HBP model was used to characterise the rheological properties of propellant slurry [47,48,49,50,51]. The HBP model, proposed by Papanastasiou, approximates the HB model using a continuous function. Later, Frigaard et al. demonstrated in their study that this model can better solve the divergence problem of the equivalent viscosity coefficient under small deformation. Equations (5) and (6) show the expression of the HBP model and its equivalent viscosity coefficient.

(5) $$\tau =\eta_{0}{\left(\gamma \right)}^{n}+\tau_{y}\left(1-e^{-m\gamma }\right)$$

(6) $$\eta =\eta_{0}{\left(\gamma \right)}^{n-1}+\frac{\tau_{y}}{\gamma }\left(1-e^{-m\gamma }\right)$$

where *η*_0_ denotes shear viscosity, *τ*_y_ denotes yield stress, *γ* denotes shear rate, and n and m are controlled parameters.

Although the HBP constitutive model can characterise the flow characteristics of non-linear Bingham fluid, transporting, pouring, or performing other processes with HTPB propellant slurry takes a long time after mixing is completed. During these processes, the internal curing reaction causes significant variations in the slurry’s rheological properties, as well as changes in various parameters in the constitutive model. If the parameters *η*_0,_ *τ*_y_, n, and m in Equations (5) and (6) are fixed, they can only describe the rheological properties of slurry at a specific moment or within a smaller time range. When the time range is extended, the actual values of fluid parameters deviate significantly from the set values, making it impossible to accurately characterise the rheological properties of the slurry at this time. To modify the HBP model and allow it to describe the variations in the rheological properties of the slurry with curing time, let

(7) $$\begin{array}{l} \eta_{0}=\eta_{0}\left(t_{s}\right);\;\tau_{y}=\tau_{y}\left(t_{s}\right);\\ n=n\left(t_{s}\right);\;m=m\left(t_{s}\right) \end{array}$$

The original equations are converted into

(8) $$\begin{array}{ll} \tau = & \eta_{0}\left(t_{s}\right){\left(\gamma \right)}^{n\left(t_{s}\right)}\\ & \;+\tau_{y}\left(t_{s}\right)\left(1-e^{-m\left(t_{s}\right)\gamma }\right) \end{array}$$

(9) $$\begin{array}{ll} \eta = & \eta_{0}\left(t_{s}\right){\left(\gamma \right)}^{n\left(t_{s}\right)-1}\\ & \;+\frac{\tau_{y}\left(t_{s}\right)}{\gamma }\left(1-e^{-m\left(t_{s}\right)\gamma }\right) \end{array}$$

Equation (9) represents the HBPW viscosity constitutive model, with t_s_ denoting the curing time of the hydroxybutylene propellant slurry. After introducing curing time as a variable, parameters such as *η*_0_, *τ*_y_, n, and m are expressed as functions of time, resulting in different values at different curing times. If the variation law of these parameters with curing time can be determined, the viscosity behaviour of the slurry over a longer time period can be more accurately described. This method provides a more detailed description of the propellant slurry’s rheological properties throughout the entire process. The values of *η*_0_, τ_y_, n, and m in the equation can be calculated and fitted using measurement data and relevant theories.

## 3. Experimental Material: HTPB Propellant Slurry

**Figure 2:**
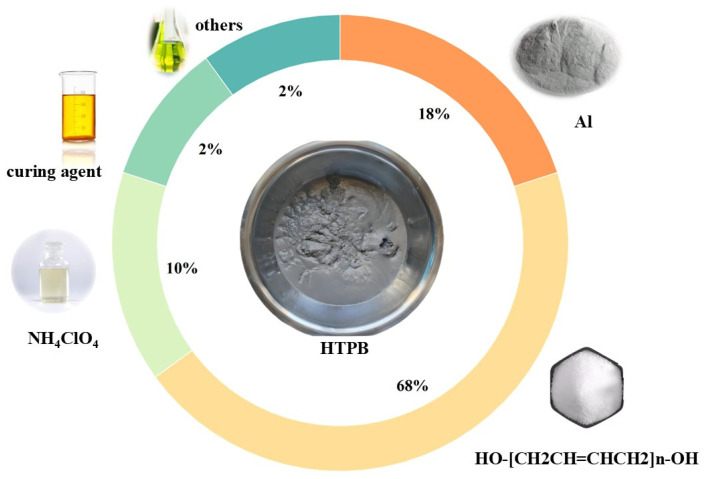
Formulation of HTPB propellant slurry.

Figure 2 depicts the compositional formula of the propellant explored in this paper. The HTPB propellant employed ammonium perchlorate (AP) as an oxidant with a molecular formula of NH_4_ClO_4_, which provides 34% of accessible oxygen; metal aluminium powder was used as fuel to increase the heat of combustion. Metal aluminium powder is abundantly available, inexpensive, and has a high calorific value; hence, it is extensively employed in the production of composite propellants. As a matrix for forming a cross-linked network structure, the HTPB precursor gives the propellant a specific shape and the ability to withstand a certain load; the curing agent works by interacting with reactive functional groups of various components in the binder system, resulting in a cross-linked polymer network structure. In addition, plasticizers are added to improve the slurry’s processing and mechanical qualities.

This study focuses on a specific HTPB propellant slurry with an 86% solid content. Figure 2 displays the component fractions.

## 4. Rheological Measurements: Instruments and Conditions

Rheological measurement uses simple flow patterns to evaluate fluid rheological response parameters such as stress and strain. In reality, the rheological response characteristics of the fluid cannot be measured directly. Conversion is required to transform measured values of pressure, rotation speed, torque, and other relevant parameters into rheological response parameters under different conditions. As a result, rheological measurement theory must establish the link between these measurable quantities and rheological response variables. The principal measuring instruments are a viscometer and a rheometer. The former is simple but has a narrow measurement range, whereas the rotary rheometer is frequently used because of its broad measurement range and good precision.

The torque of a liquid as the rotor rotates is measured by the rotary rheometer, which comes in both cone–plate and plate–plate varieties. The cone–plate rheometer has advantages in terms of constant shear rate and low sample quantity, but for multi-phase fluid systems such as HTPB propellant slurry, the plate–plate rheometer is better suited because it can measure higher shear rates, is easy to clean, and is independent of particle size.

**Figure 3:**
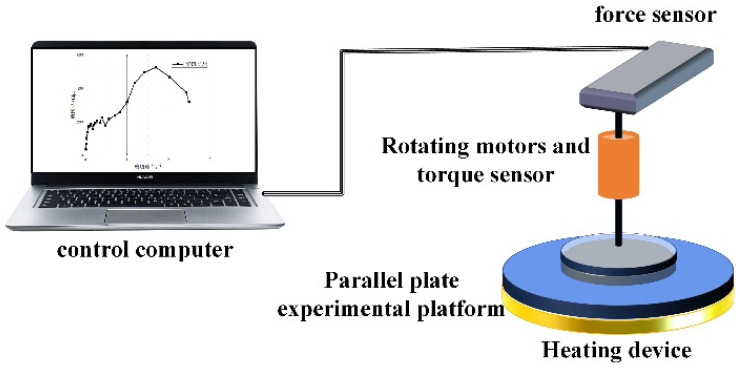
Schematic of the rotating rheometer principle.

**Figure 4:**
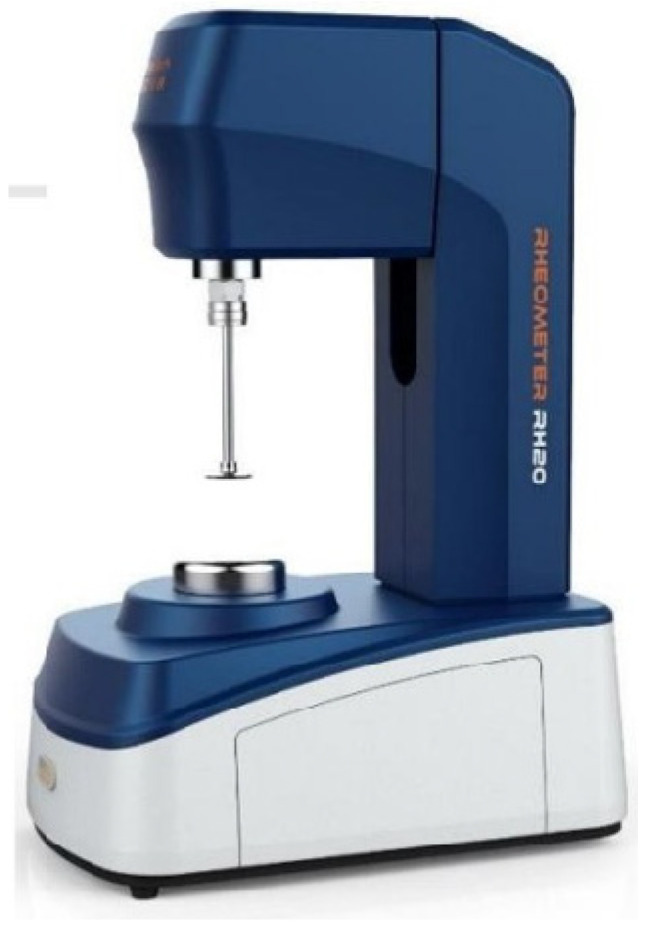
Physical image of rotary rheometer.

In actual production, the propellant slurry will be kept at a consistent temperature of 50 °C throughout the mixing, transportation, and pouring processes. The test used a rotary rheometer (Baosheng H20, Shanghai, China) to control the test temperature, and the operational software (BosinTechRH V1.1.0.1) allowed for the configuration of operating processes. Figure 3 shows a schematic diagram of the H20 rotary rheometer, whereas Figure 4 shows the actual picture.

When designing rheological testing, it is necessary to account for both curing time and shear rate. According to the process flow of mixing and pouring propellant slurry, the total time required from the end of mixing to the end of pouring is usually around 5 h. As a result, the investigation began with the completion of mixing the slurry (t = 0) and investigated the following fluctuation law of the slurry’s rheological properties over a 5 h period. The test design should take into account controlling the shear rate within a safe range, such as 0 s^−1^ to 10 s^−1^. Simultaneously, to reduce the impact of particles in the HTPB propellant slurry, the spacing between parallel plates during the test was limited to 2 mm.

## 5. Analysis of Rheological Measurement Data

Rheological measurements can be classified as steady-state, transient, or other measurement kinds based on the flow’s time dependency or deformation history. A steady-state test is a flow measurement under shear action in which the deformation rate remains constant over time; a transient rheological measurement is one in which the stress or strain rate changes abruptly. The steady-state test applies a constant shear rate to the fluid to be evaluated and measures the variation in the fluid’s apparent viscosity or shear stress over time as a result of the constant shear rate. The steady-state shear test is preferred because it clearly exposes the evolution of fluid viscosity with shear and allows for precise adjustment of rheological model parameters. Transient testing procedures use rapidly fluctuating shear rates on a liquid sample while keeping it stable in the testing equipment. This method allows for the observation of the evolution of parameters such as viscosity and shear stress in the liquid over time, as well as an in-depth analysis of the slurry’s viscosity characteristics.

### 5.1. Analysis of Steady-State Testing Data

**Figure 5:**
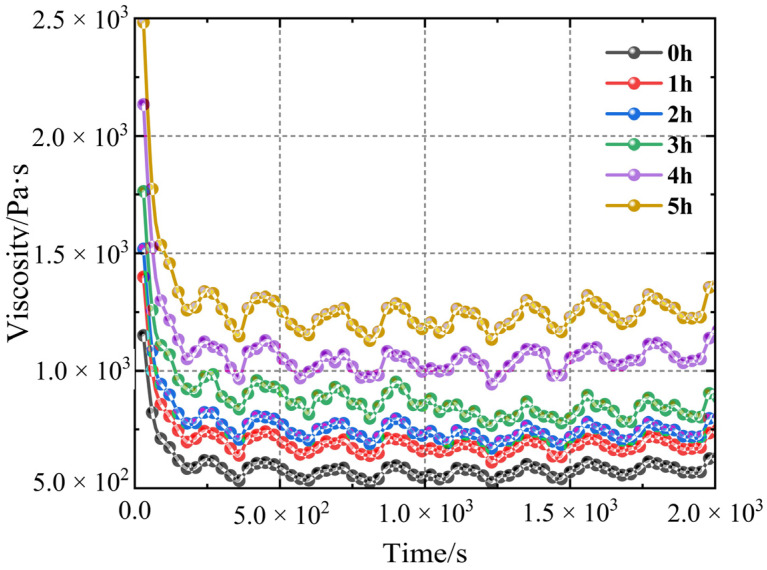
Steady-state viscosity test chart of propellant slurry with a curing time of 0 to 5 h.

Figure 5 depicts the viscosity data collected during the test at a shear rate of 1 s^−1^. The viscosity of the slurry is highest at first but steadily lowers during the shearing process. After a specific degree of viscosity is reached, there is no visible change, indicating that the propellant slurry is thixotropic. In summary, the impact of a constant shear rate on the microstructure of the slurry causes the viscosity to vary from high to low. When the curing time is 0 h, the curing reaction has not yet begun; therefore, the initial viscosity and steady-state viscosity are not significantly different. Nevertheless, the difference increases when the curing time is 5 h. Table 1 presents the statistics and comparisons.

The difference between initial and steady-state viscosity is caused by changes in the conformation of polymer chains in the slurry. Under a constant shear rate, the internal polymer chains in the slurry gradually stretch and straighten, reducing the stress generated by the rheometer and decreasing the viscosity. When the polymer chains reach a steady state, the viscosity remains constant, referred to as steady-state viscosity.

The test determined the viscosity variation over five curing times and a steady-state test lasting 2000 s. Observing the steady-state test data of the rotary rheometer with five curing times, it can be found that within 0–200 s, the test viscosity decreases linearly at each testing time, with the highest values located at 1150.28, 1403.34, 1515.61, 1753.54, 2142.86, and 2483.34 Pa·s. It follows that as the propellant slurry solidifies, its viscosity gradually increases. The viscosity fluctuates in a specific range over the next 200 s. The average viscosity in the stable stage is used to calculate the viscosity at a constant shear rate after organising the data.

Vertical analysis reveals that, after the same acting time of shearing, the apparent viscosity of the slurry increases with the length of the curing cycle. The viscosity difference grows larger as shear time increases. At the start of a shear test (when shear time is zero), the curing time ranges from 0.5 to 5 h, resulting in a viscosity difference of approximately 1000 Pa·s. After 180 s of shearing, the pressure difference exceeds 2000 Pa·s. Further calculation reveals that, under the same shear rate, the viscosity of the slurry generally decreases by about 50% during the solidification period of 0 to 5 h, with the slurry cured for 3 h showing the most significant decrease of 52.53%.

### 5.2. Analysis of Transient Test Results

Yield stress is an important rheological property for HTPB propellant slurry; it is defined as the critical stress threshold at which the fluid transitions from a solid to a liquid state. When the stress in a slurry is less than the threshold, it behaves like a solid and does not flow. If the yield stress is high, the flowability of a slurry suffers, reducing processing performance. The yield stress in a transient test varies with the shear rate. In the beginning, the relationship between shear stress and yield stress is linear, but it later becomes non-linear. The yield stress of the fluid is determined by the yield stress at the transition point, which is the boundary between linear and non-linear behaviour.

**Figure 6:**
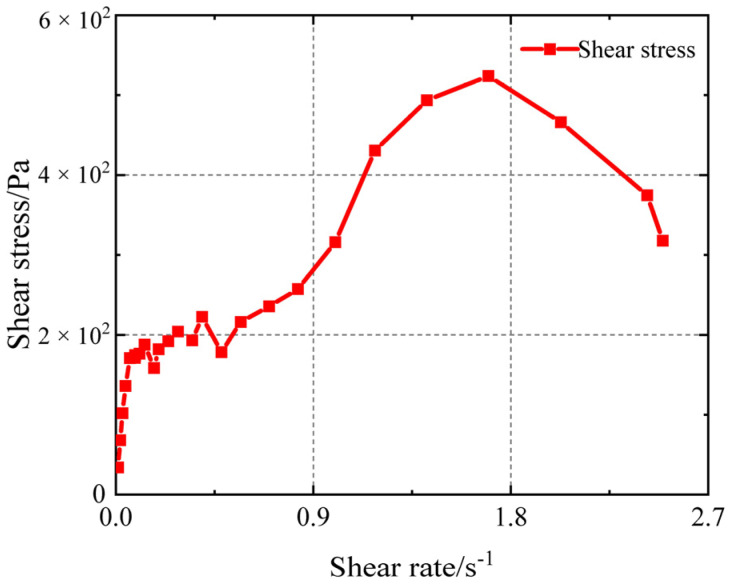
Yield stress measured by test.

Figure 6 shows the data points for a curing time of 0 h. The figure’s ordinate represents shear stress, while the abscissa represents shear rate. As the shear rate increases, the yield stress rises at a constant rate for a while before decreasing as an inflexion point appears. The yield stress of the propellant slurry, which is 178 Pa, is used to calculate the shear stress at the curve’s first inflexion point.

The yield stress of HTPB propellant slurry with varying curing times was measured using the above-mentioned testing method, with a one-hour measurement interval. Table 2 contains the final measurement results. It is evident that as the curing time increases, so does the yield stress. That is, as the curing process progresses, more external force is required to move the slurry.

**Figure 7:**
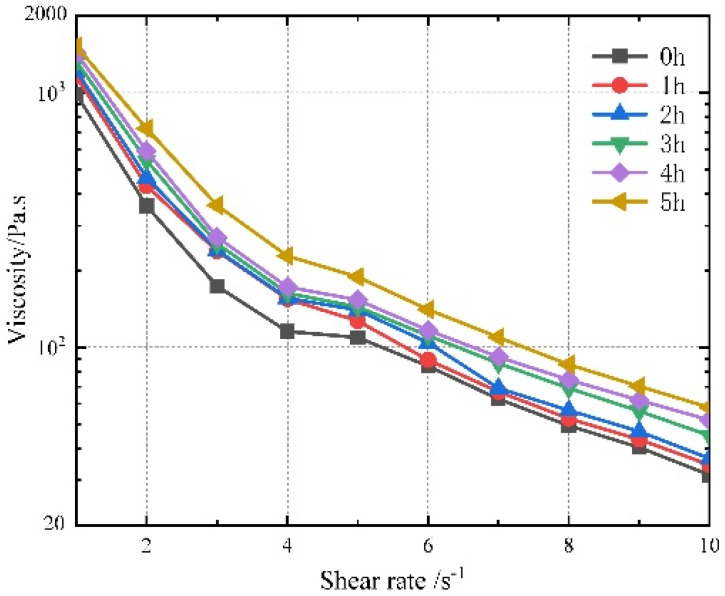
Steady-state viscosity of samples with a curing time of 0–5 h.

In addition, the test yields a viscosity that varies with shear rate, as illustrated in Figure 7. Comparing the viscosity of propellant slurry with different curing times at the same shear rate reveals that the relationship between viscosity and time is essentially non-linear. When the shear rate is less than 4 s^−1^, the viscosity varies significantly. When the shear rate is greater than 4 s^−1^, the shear rate remains stable. And the value is less than 400 Pa·s.

**Figure 8:**
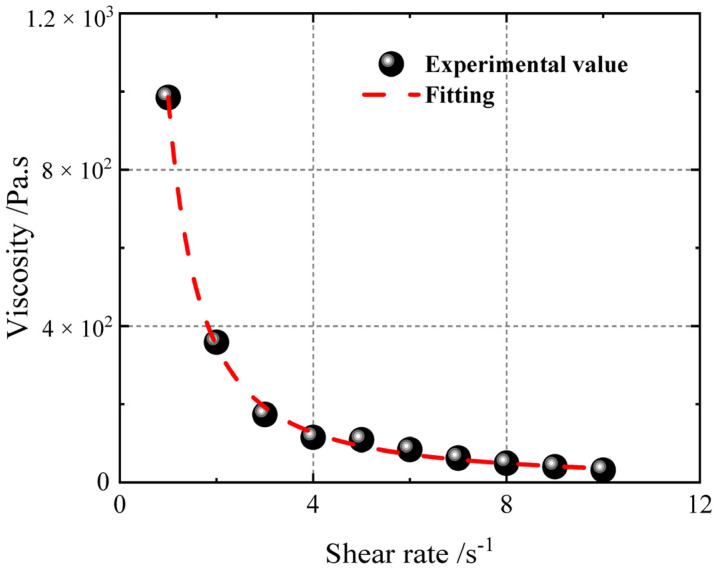
Viscosity data at different shear rates.

The variation in initial viscosity (t = 0) with shear rate is analysed and fitted using the Levenberg–Marquardt algorithm (hereinafter referred to as the LM algorithm) on the basis of the HBP constitutive model, yielding the viscosity constitutive model shown in Figure 8 below. The test results show that as the shear rate increases, the viscosity gradually decreases. When the shear rate is low, the viscosity changes dramatically.

Equation (9) is used to fit the viscosity constitutive model to the HTPB propellant slurry, with fitting parameters listed in Table 3 and a fitting accuracy of R^2^ = 0.997.

**Figure 9:**
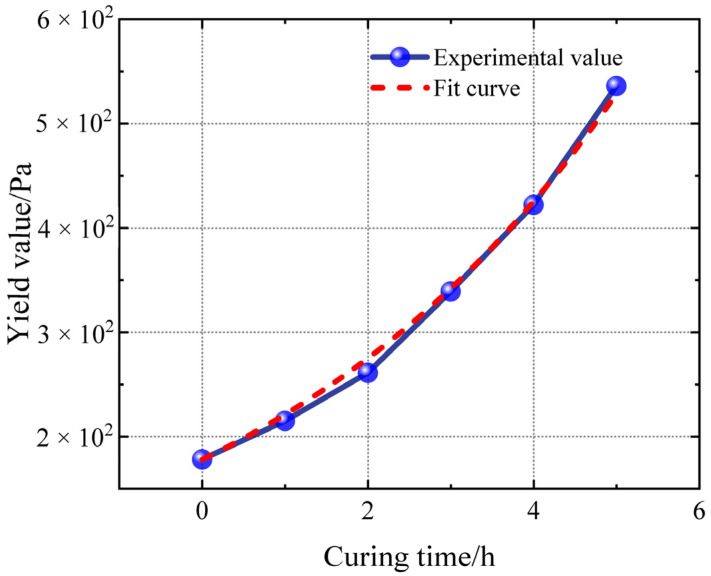
Yield stress–time curve.

The variation in yield stress over time is also evident in the blue data points presented in Figure 9. The observed yield value exhibits a logarithmic variation with curing time. The variation trend in yield value shows that after mixing, the slurry immediately exhibits an obvious yield characteristic, and the initial yield stress is relatively low. An accelerating trend can be observed in the increase in yield stress as time passes. The yield stress of the slurry increases significantly with each hour, particularly in the last two. The yield stress during the solidification stage is approximately 3.5 times the initial value after 5 h of curing. According to filling system theory, yield behaviour is strongly influenced by factors such as filler particle arrangement and agglomeration structure, as well as particle–liquid interaction. The curing reaction within the propellant slurry increases the adhesion force at the interfaces as well as the viscosity of the liquid phase, resulting in a tighter particle arrangement and a continuous increase in yield stress.

To establish the mathematical relationship between increasing yield stress and curing time, the data shown in Table 3 and Table 4 are fitted by curves. The fitting takes curing time as an independent variable and the yield stress of the slurry as a dependent variable. The non-linear fitting used the LM algorithm with the logarithm function as the target function. The red short dash line represents the curve after curing, as expressed by Equation (10).

(10) τy=A*e^(B*x)

where *A* = 178 and *B* = 0.21786, with R^2^ = 0.996, indicating a good fit result. The fitted yield stress equation is as follows:

(11) τy=178*e^(0.2186*x)

To obtain the above-mentioned HBPW model, the viscosity–shear rate constitutive model is fitted for each curing time. The viscosity of the slurry with six curing times ranging from 0 to 5 h is fitted, and the constitutive parameters for each curing time are determined, as shown in Table 4.

There are four parameters that vary based on the curing time, as shown in Equation (7) from the previous discussion, where *τ_y_* has been determined as a function of time. The other parameters have been fitted using the LM algorithm to determine the functional relationship of each parameter over time. All R^2^ values exceeded 0.99, indicating a high level of accuracy in fitting.

(12) $$\eta_{0}=a\cdot t^{2}+b\cdot t+c$$

where a = −9.328; b = 83.39; c = 829.2;

(13) n=d⋅t+f−1

where d = 0.02065 and f = −0.6561;

(14) $$m=-(g\cdot e^{-h\cdot x}+l)\cdot \gamma$$

where g = 26.23; h is −13.37; l = −22.23.

**Figure 10:**
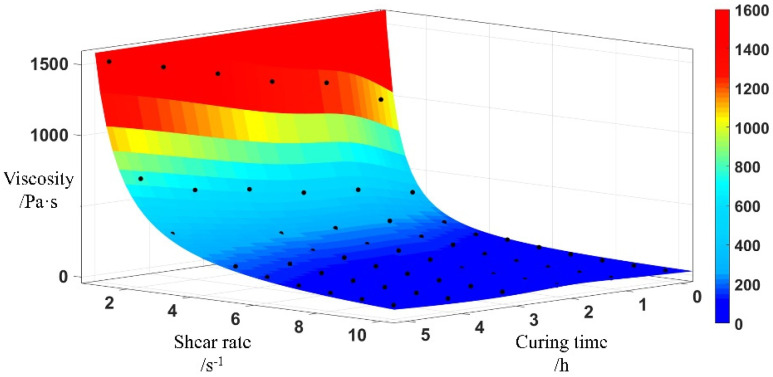
Viscosity curve.

By plugging the functions determined by the parameters in Equations (13)–(15) into Equation (7), we can derive the correlation between viscosity, curing time, and shear rate, represented by Equation (15). Based on Equation (15), it is possible to construct a three-dimensional viscosity surface of the HTPB propellant slurry using the HBPW model, as shown in Figure 10. The viscosity data obtained by the test is represented by the black dots in the figure. On this surface, you can obtain a precise viscosity value for any given curing time and shear rate. The time-adjusted HBPW model provides a more accurate representation of the rheological behaviour of propellant slurry compared to the original HBP model. In the three-dimensional space depicted in Figure 10, the shear rate, curing time *t*_s_, and slurry viscosity collectively create a three-dimensional viscosity surface. Each specific point on the curing time corresponds to a plane that is parallel to the surface.

On this surface, a precise viscosity can be determined for any given curing time and shear rate.

(15) $$\eta =(a\cdot x^{2}+b\cdot x+c)\cdot \gamma^{\left(d\cdot x+f-1\right)}+(\frac{178\cdot e^{0.2186\cdot x}}{\gamma })\cdot (1-e^{-(g\cdot e^{-h\cdot x}+l)\cdot \gamma })$$

## 6. Rotary Rheometer Simulation Based on Finite Volume Method

Simulation analysis is employed to model the flow of propellant slurry with different curing times of 0 h and 4 h through a hole in order to assess the influence of solids on the pouring process.

In this section, we explore the finite volume method combined with the volume of fluid method (VOF) to conduct comprehensive simulation research on the operation process of a rotary rheometer. The section primarily discusses simulation analysis under a specific condition, where a constant shear rate of 1 s^−1^ is used. This allows for a careful simulation of the rheological behaviour at this particular shear rate.

### 6.1. Pre-Processing Process

**Figure 11:**
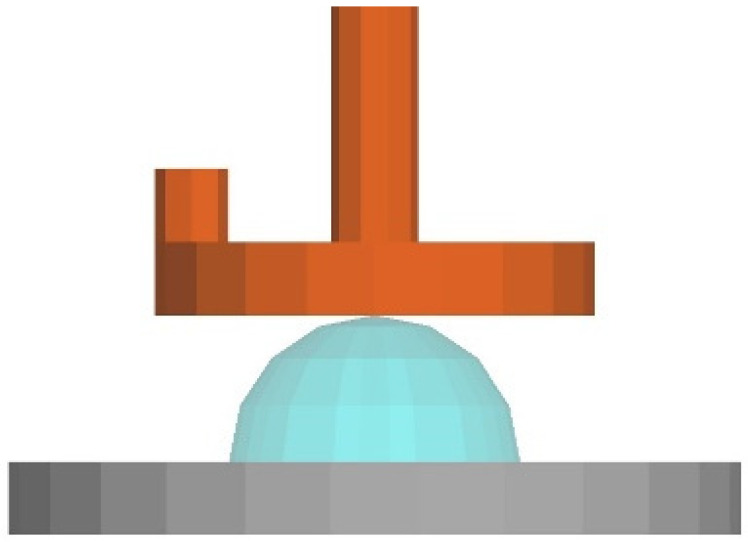
Three-dimensional simulation model of rotary rheometer.

A finite element simulation model was established using the dimensions of the clamp used in the test. During the test, a parallel plate clamp with a diameter of 20 mm was utilised to conduct the rheological test. As the rotor spins, the gap between the parallel plates measures 2 mm. The upper plate had a diameter of 20 mm, while the lower plate had a diameter of 30 mm. The distance between the upper and lower parallel plates started at 15 mm and decreased to 2 mm, as shown in Figure 11. The movement of parallel plates is divided into two stages of equal size. The upper plate drops from 15 mm to 2 mm in the first stage and then moves at a shear rate of 1 s^−1^ in the second stage. Since the rheometer used in the experiment measures the shear rate of the fluid at the 2/3 radius of the parallel plate, we set the rotational speed of the upper plate in the second stage to 2.3 rad/s.

**Figure 12:**
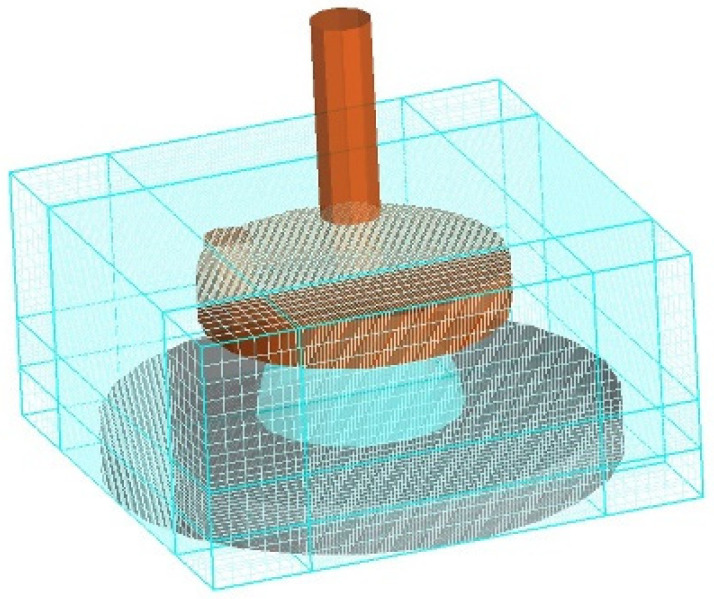
Grid diagram simulated.

The central area has been divided into smaller sections to improve calculation efficiency. The division is performed by adjusting the number of grids in the x, y, and z directions. The x and z directions are both horizontal directions, while the y direction is vertical. The grids in the grid are presented as cubes, with equal dimensions in the x and y directions (150 grid layers) and a smaller dimension in the z direction (50 grid layers). The centre grid and edge grid have the same size, with the centre grid measuring 0.0002 mm and the edge grid measuring 0.002 mm, as shown in Figure 12.

**Figure 13:**
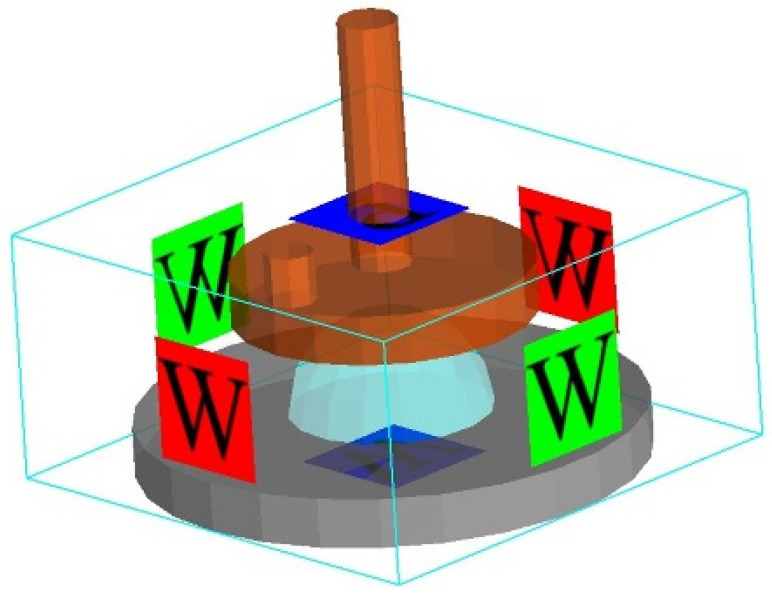
Simulated boundary conditions.

To accurately simulate the real environment during the test, the pressure above the surface must be set to the standard atmospheric pressure (P). Other parts are set as the wall surface: W. The boundary conditions are set as illustrated in Figure 13.

### 6.2. Solving Method

In computational fluid dynamics, the finite volume method (FVM) offers a strong numerical basis for accurately solving fluid dynamics equations due to its exceptional conservation properties and ability to handle intricate geometric shapes. VOF, on the other hand, greatly improves the accuracy of simulations on free surfaces and multiple intersecting interactions. This is because of its efficient tracking of interfaces in multi-phase flow and its precise capture of the evolution of complex interfaces. By combining these two methods, it is possible to ensure the conservation of physical quantities and handle complex topological changes in multi-phase flow without the need for a pre-griding process on interfaces. This greatly enhances the flexibility and practicality of numerical simulation. This approach offers an accurate and effective numerical solution strategy for addressing multi-phase flow problems in engineering and scientific research.

#### 6.2.1. VOF Method

The simulation uses the VOF method, which means that the momentum equations apply to all fluid components. The phase volume fraction F is used as a variable to track the interface within the computational domain. The volume fraction of one phase in the grid is represented by F.

The model simulates two or three immiscible fluids by solving the momentum equation and managing the volume fraction of each fluid. Common uses of the model involve fluid injection, movement of bubbles, dam flow, and gas–liquid interface treatment. VOF is primarily suited for non-steady multi-phase flow models and is limited to specific steady-state problems. The interface is constructed by calculating the phase fraction of grid cells.

#### 6.2.2. Finite Volume Method

The simulation process is conducted using the finite volume method. Typically, there are multiple approaches available for solving the partial differential equations of fluid problems, such as the finite difference method (FDM), the finite element method (FEM), the spectral method, and the meshless method. The primary objective of these methods is to estimate the analytical solutions of continuous partial differential equations using a discrete system of equations.

The finite volume method is a numerical calculation method that offers high computational accuracy, wide applicability, and improved computational efficiency. The average value within the controlled volume is utilised to describe physical quantities, thereby avoiding the interpolation errors commonly encountered in FEMs. It is also well-suited for unstructured meshes, making it ideal for tackling complex problems involving free surfaces, like the pouring of slurry.

### 6.3. Analysis of Simulation Results

**Figure 14:**
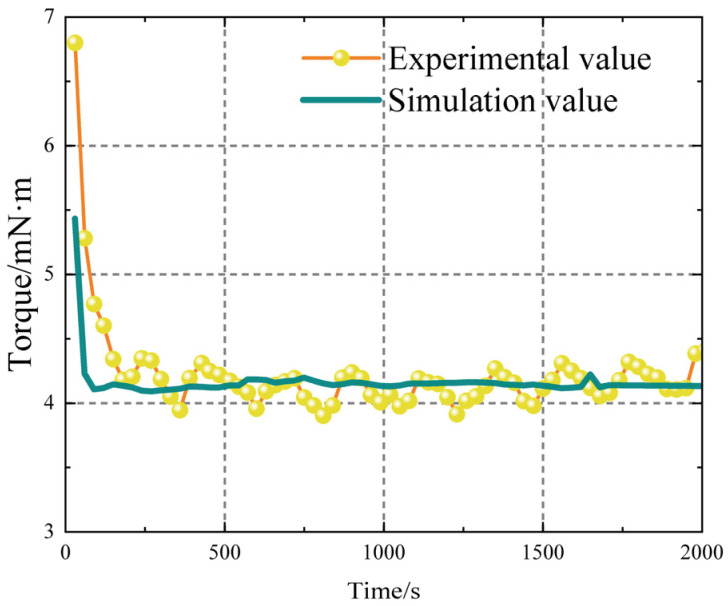
Comparison of experimental and simulated torque on the upper parallel plate.

The torque experienced by the upper parallel plate in the simulation was extracted and compared with the torque obtained from the test. Figure 14 displays the data. The simulation is capable of accurately replicating the test process. The data remains consistent throughout the simulation due to the stable environmental settings. The calculated average value of the tested and simulated torque stabilisation stages reveals a simulation error of 1.75%. The HBPW viscosity constitutive model accurately describes propellant viscosity.

The test data experience periodic fluctuations. This is because during the testing process, the traditional Chinese medicine slurry undergoes aggregation, forming micro clusters. Concurrently, the diameter of these micro clusters gradually increases. However, once the diameter reaches a certain threshold, the clusters disperse under shear action. This behaviour suggests that the high solid content in the propellant slurry leads to unique linearity characteristics in the HTPB propellant slurry. The formation of micro clusters also implies that cone–plate rheometers are unsuitable for such tests due to their variable spacing, which can interfere with the test data of micro clusters. Therefore, the use of plate–plate rheometers is more appropriate. Additionally, the simulation did not account for the influence of the particle phase, resulting in data that remained largely stable without significant fluctuations.

Observing Figure 14, it is apparent that at the initial time, the experimental torque is higher than the simulated torque. This discrepancy arises because the initial slurry contains some particles in an agglomerated state. When the rheometer shears these agglomerated particles, it exerts a higher torque due to the disruptive effects on the agglomerates. In contrast, the simulation does not account for these particles, resulting in a lower simulated torque at the initial moment. Thus, the experimental torque is higher than the simulated torque at the beginning.

**Figure 15:**
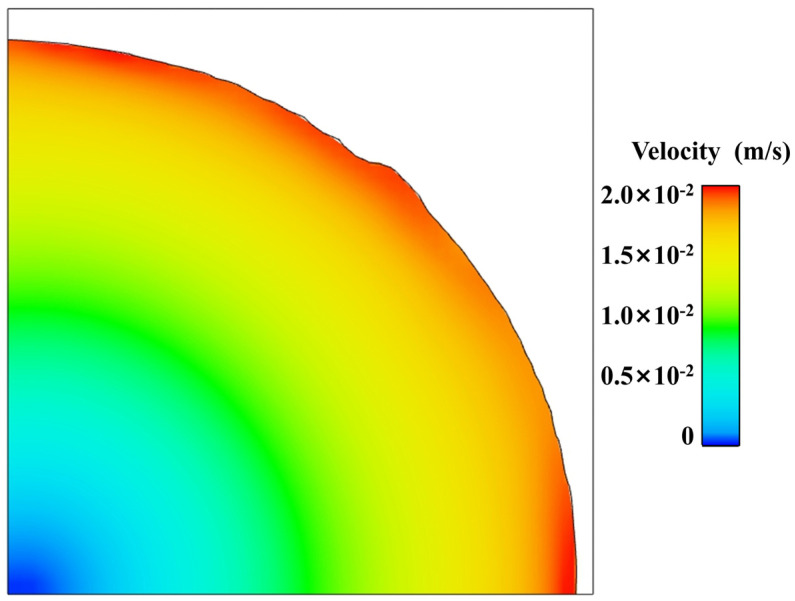
Cloud map of velocity at cross-section.

Figure 15 displays a velocity cloud map of the 1.1 mm section, with arrows indicating the flow direction of the propellant slurry. It is evident from observing the flow process that the fluid moves in the same direction as the top parallel plate. Additionally, the slurry velocity at the edges is higher, reaching 0.02 m/s, while the velocity at the centre is nearly 0.

**Figure 16:**
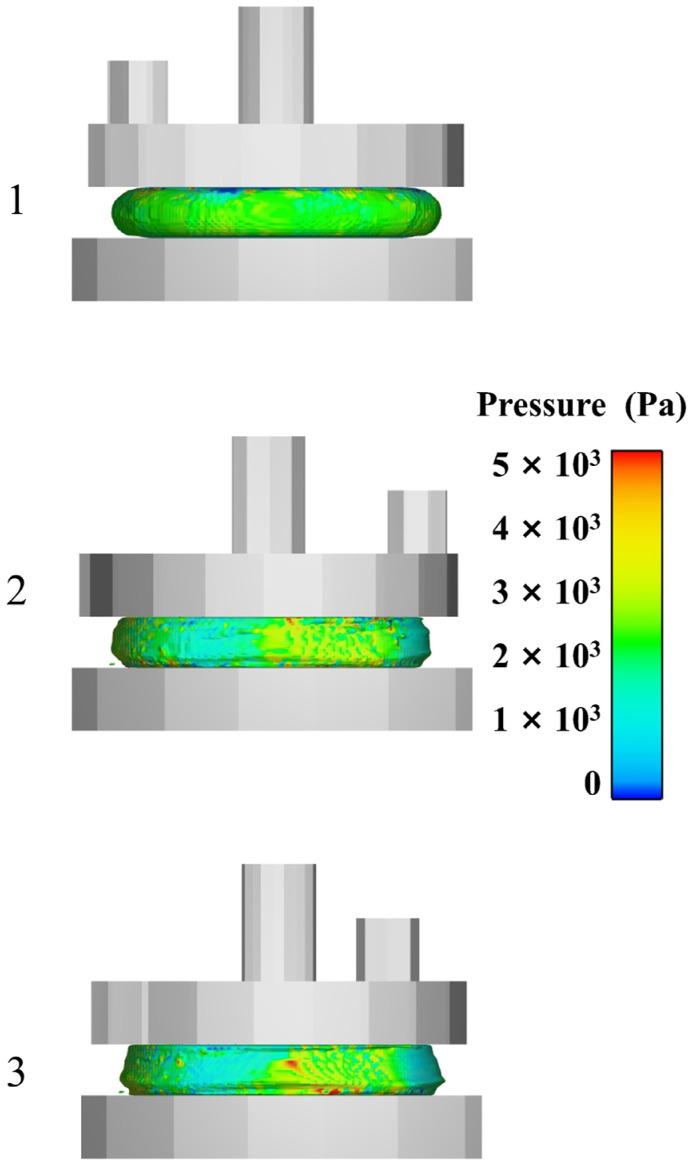
Simulated side view.

**Figure 17:**
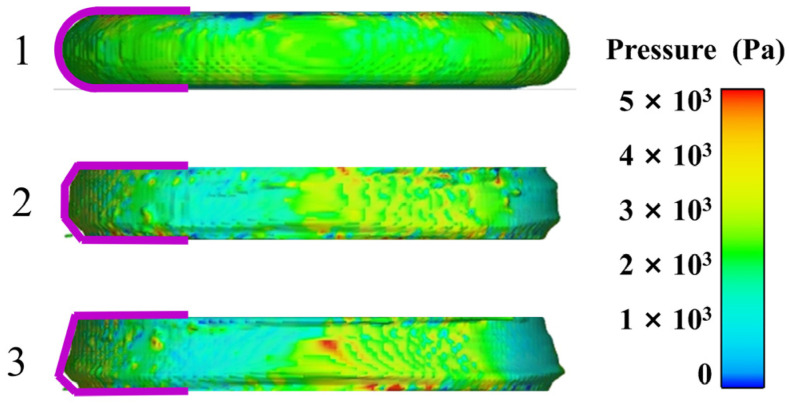
Simulated boundary shapes.

Figure 16 displays the different rotational stages of the upper parallel plate, illustrating the progression from early to middle to late stages, arranged from top to bottom. Here, No. 1 represents the early stage, No. 2 the middle stage, and No. 3 the late stage, corresponding to the initial, developmental-extreme, and stable stages of the flow field variation, respectively. Figure 17 provides a detailed view, illustrating the progression from start to finish. As the upper parallel plate falls, the propellant slurry gets compressed. The cloud map observation in the early stage reveals a gradual approach to the slurry’s edge, forming a smooth arc. Concurrently, the pressure field remains consistent, and there are no abrupt pressure fluctuations, suggesting that there is no occurrence of wide-scale flow in the slurry. Based on the cloud map in the middle state, it is evident that the rotation of the parallel plate causes internal flow and leads to non-uniform pressure. The slurry flows inside under the action of parallel plates. The uppermost and lowermost slurries adhere to the plate’s surface, with the middle portion gradually bulging out due to centrifugation. Concurrently, the slurry’s edges are clearly visible, while the height in the middle stands out. Based on the cloud map of the slurry in the late stage, it is evident that the pressure distribution becomes less uniform and there is an increase in high-pressure areas. In addition, the positions with protruding edges shift and gradually move closer to the lower parallel plate. This occurs because the propellant slurry naturally moves downward due to gravity, causing its shape to gradually resembles a cone with a protruding corner at the edges.

**Figure 18:**
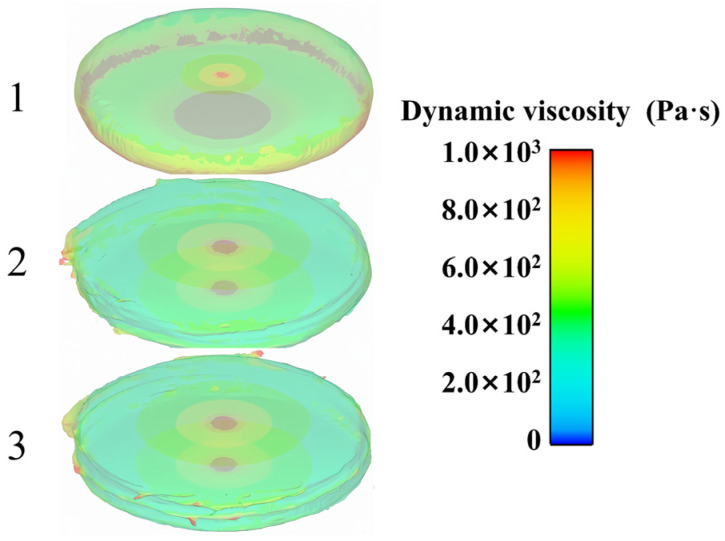
Cloud map of viscosity.

Figure 18 displays the cloud map of the slurry’s viscosity, showing the different rotational stages of the parallel plate at various points in time. At the beginning, there is no internal flow within the slurry and no significant shearing effect across a large area, indicating the presence of areas with higher viscosity. Once the rotor has completed a rotation, the slurry inside achieves a consistent and steady state, suggesting that the internal viscosity remains constant. Concurrently, the torque on the upper parallel plate remains constant, aligning with the test data from the previous part.

## 7. Conclusions

This research consists of three main parts: investigating the constitutive model for viscosity, conducting tests on rheological properties, and verifying the findings through simulation.

1. The viscosity of HTPB propellant slurry changes as it cures over time. The functional relationship between viscosity, shear rate, and curing time was accurately described by introducing the time variable into the HBP model, resulting in the establishment of the HBPW viscosity constitutive model.

2. The rheological test results show that the viscosity of the slurry is influenced by the shear rate and curing reaction, and these influences are non-linear. There is a relationship between the shear rate and the viscosity of the slurry. Additionally, prolonging the curing time will result in a higher slurry viscosity.

3. The experimental and simulation results confirm that the HBPW viscosity constitutive model accurately represents the rheological behaviour of the slurry as it cures. In comparison to the test results, the error is a mere 1.7525%. During the test, the slurry’s morphology will be influenced by both shear and gravity.

## Figures and Tables

**Table 1:** Average steady-state viscosity of propellant slurry with a curing time of 0 to 5 h.

Curing Time /h	Initial Viscosity *η*_0_/Pa·s	Steady-State Viscosity *η*_∞_/Pa·s	*η*_0_-*η*_∞_ /Pa·s	Reducing Magnitude
0	1150.28	588.63	561.65	48.83%
1	1403.34	701.93	701.41	49.98%
2	1515.61	745.30	770.31	50.83%
3	1753.54	832.32	921.22	52.53%
4	2142.86	1094.83	1048.03	48.91%
5	2483.34	1269.47	1213.87	48.88%

**Table 2:** Yield stresses at different curing time.

Curing Time/h	Yield Stress/Pa	Rising Percent of Yield Stress
0	178	0.00%
1	215	20.79%
2	261	46.63%
3	339	90.45%
4	422	137.08%
5	536	201.12%

**Table 3:** Viscosity and constitutive parameters of slurry without curing.

*η*_0_	n	*τ*_y_	m
178.982	−0.467	170.880	−0.169

Here, *η*_0_ denotes shear viscosity; *τ*_y_ denotes yield stress; *γ* denotes shear rate; and n and m are control factors.

**Table 4:** Average steady-state viscosity of slurry with a curing time range of 0–5 h.

Curing Time/h	η_0_	n	τ_y_	m
0	971.385	−0.543	178	0.090
1	1163.473	−0.407	215	−0.038
2	1226.095	−0.408	261	−0.034
3	1342.186	−0.378	339	−0.050
4	1445.422	−0.379	422	−0.054
5	1561.055	−0.187	536	−0.145

## Data Availability

The data used to support the findings of this study are available from the corresponding author upon request.
